# Enhancement of homology-directed repair with chromatin donor templates in cells

**DOI:** 10.7554/eLife.55780

**Published:** 2020-04-28

**Authors:** Grisel Cruz-Becerra, James T Kadonaga

**Affiliations:** Section of Molecular Biology, University of California, San DiegoLa JollaUnited States; Weill Cornell MedicineUnited States; University of California, DavisUnited States

**Keywords:** chromatin, homology-directed repair, precise genome editing, Human

## Abstract

A key challenge in precise genome editing is the low efficiency of homology-directed repair (HDR). Here we describe a strategy for increasing the efficiency of HDR in cells by using a chromatin donor template instead of a naked DNA donor template. The use of chromatin, which is the natural form of DNA in the nucleus, increases the frequency of HDR-edited clones as well as homozygous editing. In addition, transfection of chromatin results in negligible cytotoxicity. These findings suggest that a chromatin donor template should be useful for a wide range of HDR applications such as the precise insertion or replacement of DNA fragments that contain the coding regions of genes.

## eLife digest

Genome editing is a powerful tool used across a wide range of biomedical research. There are several different techniques used, depending on the type of edit being made, and one known as homology-directed repair – or HDR for short – is a common technique for precisely inserting large sections of DNA, such as those needed to make desired proteins in cells.

HDR takes advantage of the cell’s mechanisms for repairing damage to DNA if both strands of the DNA double helix are broken. The mechanism relies on a DNA template to stitch the strands back together. To insert or replace a new DNA sequence, scientists can add a customized piece of DNA of their choosing to the cell so that it might be incorporated into the genome. However, HDR is not very efficient, and the success rate is often less than a few percent.

In HDR gene editing, the DNA template is typically added as purified, or ‘naked’, DNA. However, the natural form of DNA in cells, known as chromatin, is where the DNA helix is wrapped around a cluster of proteins known as histones. Cruz-Becerra and Kadonaga tested the idea that DNA in the form of chromatin might be more effective as a template for HDR than naked DNA.

The two approaches were compared to see which was better at inserting a sequence at three different locations in the genome of lab-grown human cells. In these experiments, the chromatin templates were 2.3- to 7.4-fold more efficient than the naked DNA. Also, the DNA in human cells is organized as pairs of chromosomes, and chromatin was better than naked DNA for editing both copies of the chromosome pairs rather than only one of them. In addition, the chromatin is potentially less toxic to the cells. Cruz-Becerra and Kadonaga hope that this will be useful for increasing the success rate of HDR experiments and potentially other methods of gene editing in the future.

## Introduction

The ability to manipulate genomes precisely is revolutionizing the biological sciences (Doudna, 2020). Of particular utility is the modification or insertion of customized DNA sequences at a specific genomic location by homology-directed repair (HDR) (Jasin and Rothstein, 2013). For genome engineering in cells, HDR typically involves the generation of a specifically targeted DNA double-strand break (DSB) in the presence of a homologous DNA donor template that contains the desired sequence to be modified or inserted (Urnov et al., 2005; Bedell et al., 2012; Jinek et al., 2012; Cong et al., 2013; Pickar-Oliver and Gersbach, 2019).

A key challenge in successful genome editing has been the low efficiency of HDR (Carroll, 2014; Harrison et al., 2014). For the generation of specific alterations in a short stretch of DNA (<50 nt), recently developed techniques such as base editing (Rees and Liu, 2018; Molla and Yang, 2019) and prime editing (Anzalone et al., 2019) have been shown to be highly effective. In addition, for the imprecise insertion of larger DNA fragments, homology-independent approaches can be used (Auer et al., 2014; He et al., 2016; Suzuki et al., 2016). These powerful methods cannot, however, be used for the precise insertion or replacement of >50 bp DNA fragments, such as those containing the coding regions of genes. For such applications, we considered a different strategy for increasing the efficiency of HDR in cells. Based on our previous observation that homologous strand pairing, an early step in HDR, occurs more efficiently with a chromatin donor template than with a plain (naked) DNA donor template in vitro (Alexiadis and Kadonaga, 2002), we postulated that HDR in cells might similarly be more efficient with a chromatin relative to a naked DNA donor template.

In this study, we tested this idea by comparing the efficiency of HDR with chromatin versus naked DNA donor templates in conjunction with DSBs generated by the clustered regularly interspaced short palindromic repeats (CRISPR)-Cas9 system. We found that the overall HDR efficiency as well as the frequency of homozygous editing is enhanced by the use of a chromatin donor template relative to a DNA donor template. We thus envision that a chromatin donor template, which resembles the natural form of DNA in the nucleus, could be widely used to increase the success of HDR-mediated applications, particularly those that involve the targeted insertion of DNA fragments such as the coding regions of genes.

## Results

**Figure 1.**
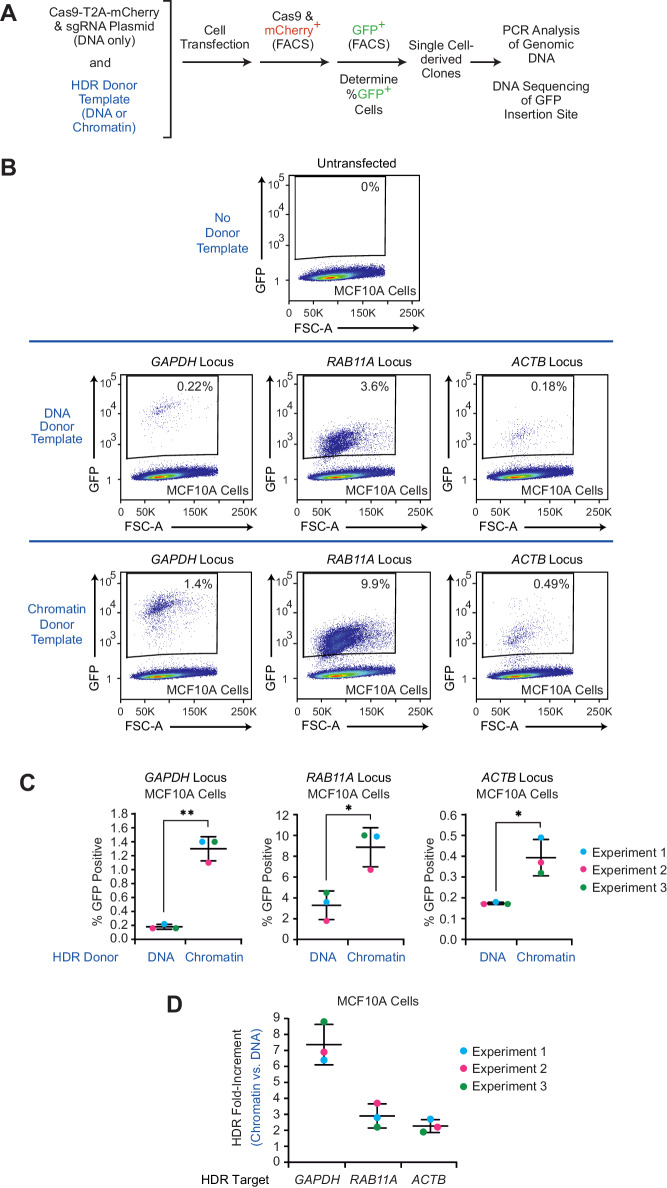
The efficiency of HDR-mediated gene editing with CRISPR-Cas9 is higher with chromatin donor templates than with DNA donor templates. (**A**) Schematic outline of the workflow in the CRISPR-Cas9-mediated editing experiments with DNA or chromatin donor templates. The HDR-mediated insertion of the GFP sequence was directed to different loci as follows. Plasmid DNA containing the coding sequence for Cas9-T2A-mCherry and a target-specific sgRNA sequence was co-transfected into different human cell lines with the corresponding HDR donor template as either DNA or chromatin. At 24 hr post-transfection, mCherry-positive cells were enriched by FACS and cultured for an additional 10 days. The expression of GFP was then analyzed by flow cytometry, and individual GFP-positive cells were sorted by FACS to generate independent clones. To determine whether there was partial or complete conversion of the multiple chromosomes containing the target genes, genomic DNA samples from each of several independent GFP-positive clones were analyzed by PCR. In addition, the precise integration of the GFP sequence at the target sites in representative edited clones was confirmed by DNA sequencing. These experiments were performed under standard CRISPR-Cas9 genome-editing conditions, as in Ran et al., 2013. (**B**) Flow cytometry analysis reveals an increase in GFP-positive cells with chromatin relative to DNA donor templates. HDR experiments were performed, as outlined in A with MCF10A cells and *GAPDH*, *RAB11A*, or *ACTB* donor templates. The population of GFP-positive cells was gated based on control cells that show no GFP expression (no donor template; upper panel; see also Figure 1—figure supplement 3). Representative data from one out of three independent experiments are shown. The results of the other two biological replicates are in Figure 1—figure supplement 4. The percentage of GFP-positive cells is indicated in each plot. FSC-A: forward scatter area. (**C**) Individual results from three independent experiments with each of the target loci. The data points from each independent experiment are designated with the same colored dots. The mean and standard deviation are indicated for each set of experiments. The *p*-values were determined by using Welch's t test. **, *p *<0.01; *, *p *<0.05. The calculated *p*-values are as follows: *p *= 0.0062 for the *GAPDH* data set; *p *= 0.017 for the *RAB11A* data set; *p *= 0.048 for the *ACTB* data set. (**D**) The use of chromatin relative to naked DNA donor templates results in a 2.3- to 7.4-fold enhancement of GFP-positive cells. The data for each of three independent HDR experiments with each locus are shown. The bars represent mean and standard deviation for each locus.

To ascertain whether the use of chromatin donor templates affects the efficiency of HDR in cells, we reconstituted three DNA donor templates (corresponding to the human *GAPDH*, *RAB11A*, and *ACTB* loci) into chromatin and tested the relative efficiencies of the targeted insertion of the GFP coding sequence with chromatin versus naked DNA versions of these templates (Figure 1 and Figure 1—figure supplements 1–4). The chromatin was reconstituted by using salt dialysis methodology with plasmid DNA and purified core histones from *Drosophila* embryos, which contain a broad mixture of covalent modifications that have not been precisely resolved (Levenstein and Kadonaga, 2002). With standard CRISPR-Cas9 methodology and human MCF10A cells (non-tumorigenic epithelial cells derived from human mammary glands), we observed that the use of a chromatin donor template relative to a naked DNA donor template resulted in a 7.4-, 2.9-, and 2.3-fold increase (average of three biological replicates) in the directed insertion of GFP sequences at the *GAPDH*, *RAB11A*, and *ACTB* loci, respectively (Figures 1B, C and D and Figure 1—figure supplements 3 and 4). Thus, at three different loci (*GAPDH*, *RAB11A*, and *ACTB*) in human MCF10A cells, there was a higher efficiency of HDR-mediated GFP insertion with chromatin donor templates than with naked DNA donor templates.

**Figure 1—figure supplement 1.**
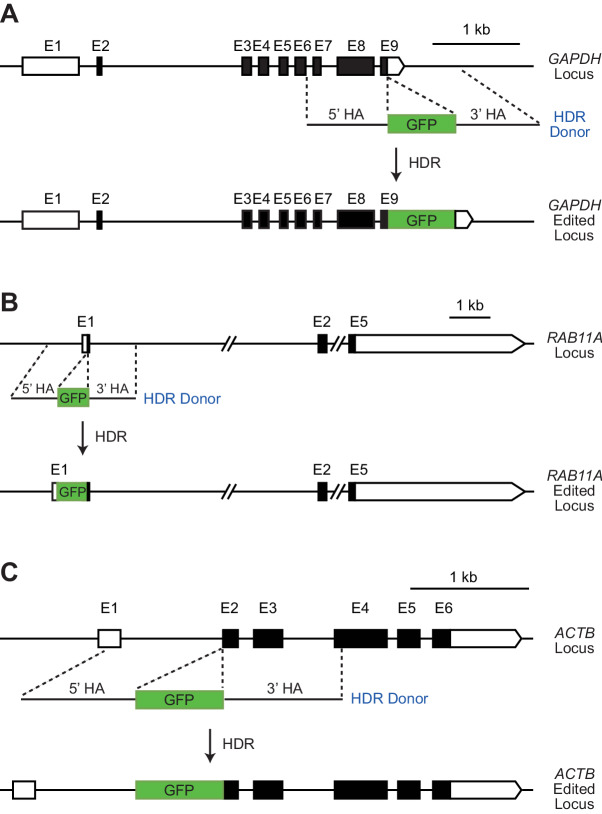
Schematic representations of the CRISPR-Cas9 target regions for HDR-mediated insertion of a GFP reporter sequence. (**A**) *GAPDH* locus. A DNA sequence that encodes the T2A self-cleaving peptide fused to the GFP protein (T2A-GFP, indicated in the figure as ‘GFP’) is inserted in exon 9 (E9) of the *GAPDH* locus. This results in the production of a GAPDH-T2A-GFP polypeptide that is spontaneously cleaved into separate GAPDH and GFP proteins. (**B**) *RAB11A* locus. The GFP sequence is inserted in the first exon (E1) of the *RAB11A* locus. This in-frame HDR-mediated insertion yields a GFP-RAB11A fusion protein. (**C**) *ACTB* locus. The monomeric enhanced GFP sequence (mEGFP; indicated as ‘GFP’) is inserted into the second exon (E2) of the *ACTB* locus. This in-frame HDR-mediated insertion results in a mEGFP-ACTB fusion protein. All three donor repair templates contain the desired insert sequence flanked by two homology arms of about 1 kb each. The dashed lines indicate the regions of homology between the HDR donor templates and the CRISPR-Cas9 targeted loci. The black boxes represent coding regions, and white boxes represent untranslated regions. E, exon; HA, homology arm.

**Figure 1—figure supplement 2.**
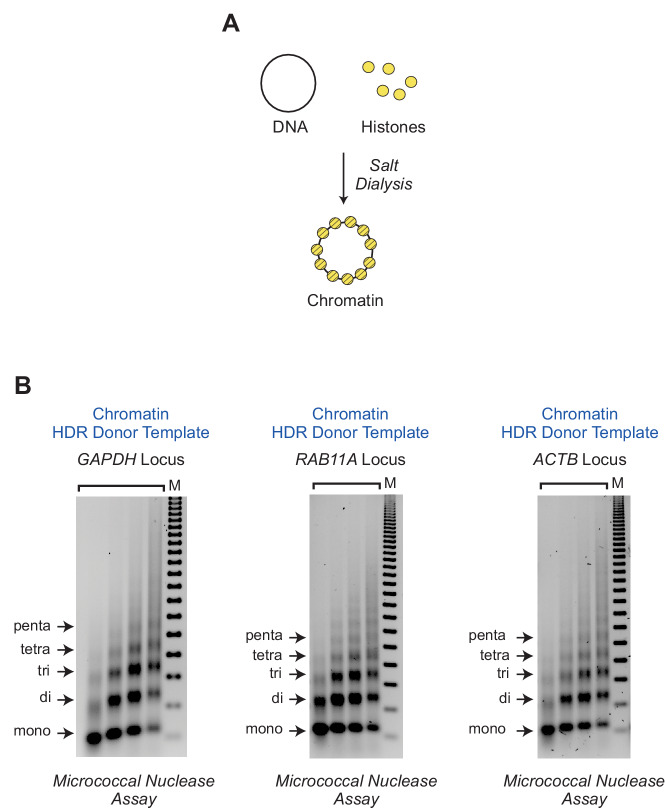
Reconstitution of plasmid DNA donor templates into chromatin. (**A**) Salt dialysis reconstitution of chromatin. The HDR donor template plasmids were reconstituted into chromatin with purified core histones by the salt dialysis method. (**B**) Micrococcal nuclease digestion analysis of chromatin reconstituted with purified components. Preparations of chromatin that were reconstituted with each of the HDR donor template plasmids (which correspond to the *GAPDH*, *RAB11A*, and *ACTB* loci) were subjected to partial digestion with four different concentrations of micrococcal nuclease. The samples were deproteinized, and the resulting DNA fragments were resolved by agarose gel electrophoresis and visualized by staining with ethidium bromide. The arrows indicate the DNA bands that correspond to mono-, di-, tri-, tetra-, and pentanucleosomes. The DNA size markers (M) are the 123 bp ladder (Millipore Sigma).

**Figure 1—figure supplement 3.**
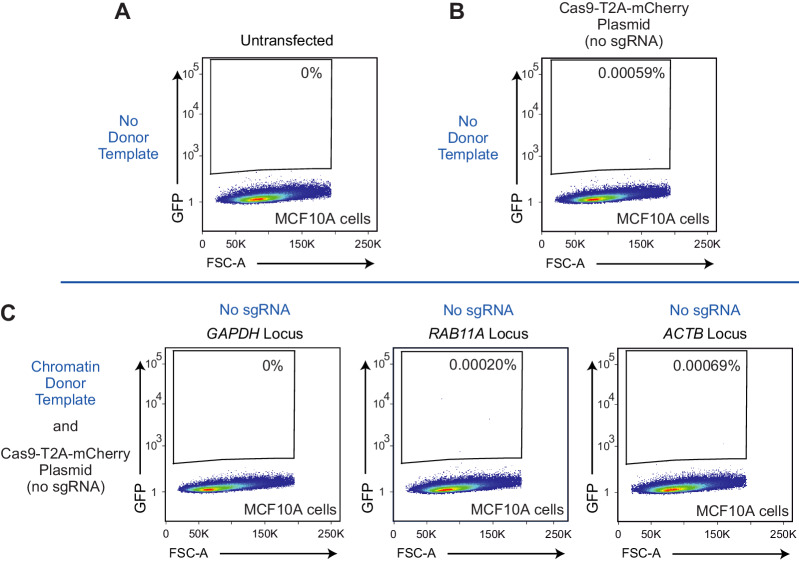
Flow cytometry analysis of MCF10A cells in control experimental conditions. (**A**) Untransfected cells. (**B**) Cells were transfected with a Cas9-T2A-mCherry plasmid (lacking an sgRNA) in the absence of a donor template. (**C**) Cells were transfected with a Cas9-T2A-mCherry plasmid (lacking an sgRNA) in the presence of the indicated chromatin donor templates. GFP positive cells in B and C, were gated based on control cells that do not contain the GFP sequence (untransfected cells). The percentage of GFP-positive cells is indicated in each plot. Representative data from one out of three experiment is shown. FSC-A: forward scatter area.

**Figure 1—figure supplement 4.**
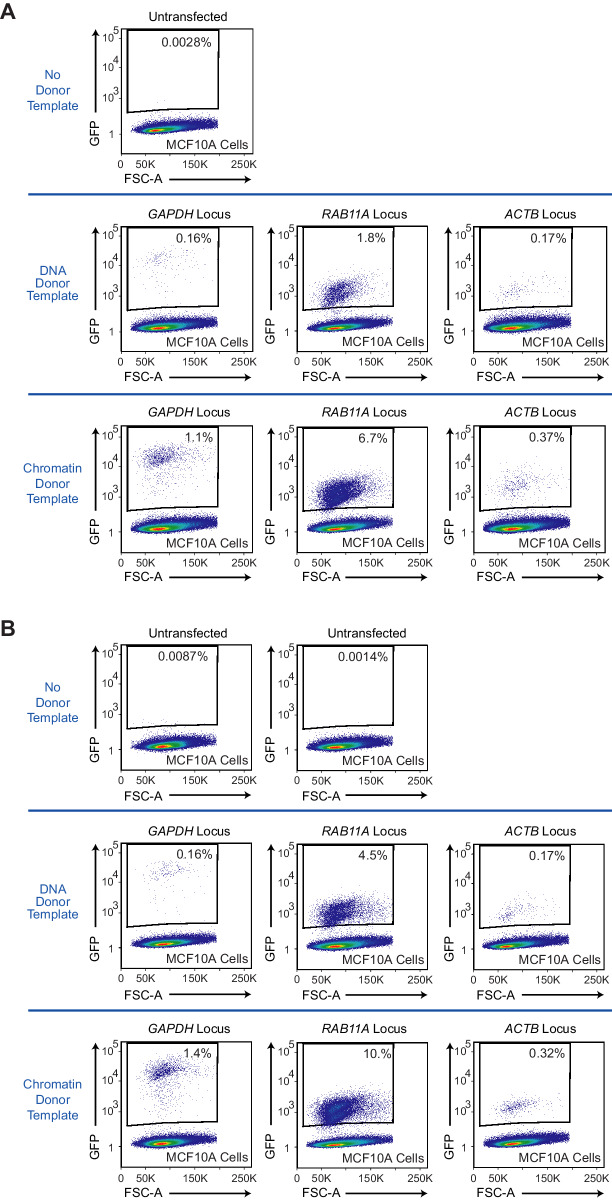
Flow cytometry analyses of biological replicates of HDR-mediated gene integration experiments in MCF10A cells. (**A**) Data from HDR experiment two with *GAPDH*, *RAB11A*, or *ACTB* donor templates. (**B**) Data from HDR experiment three with *GAPDH*, *RAB11A*, or *ACTB* donor templates. HDR experiments were performed as outlined in Figure 1A. GFP-positive cells were gated based on control cells that show no GFP expression (no donor template condition).

**Figure 2.**
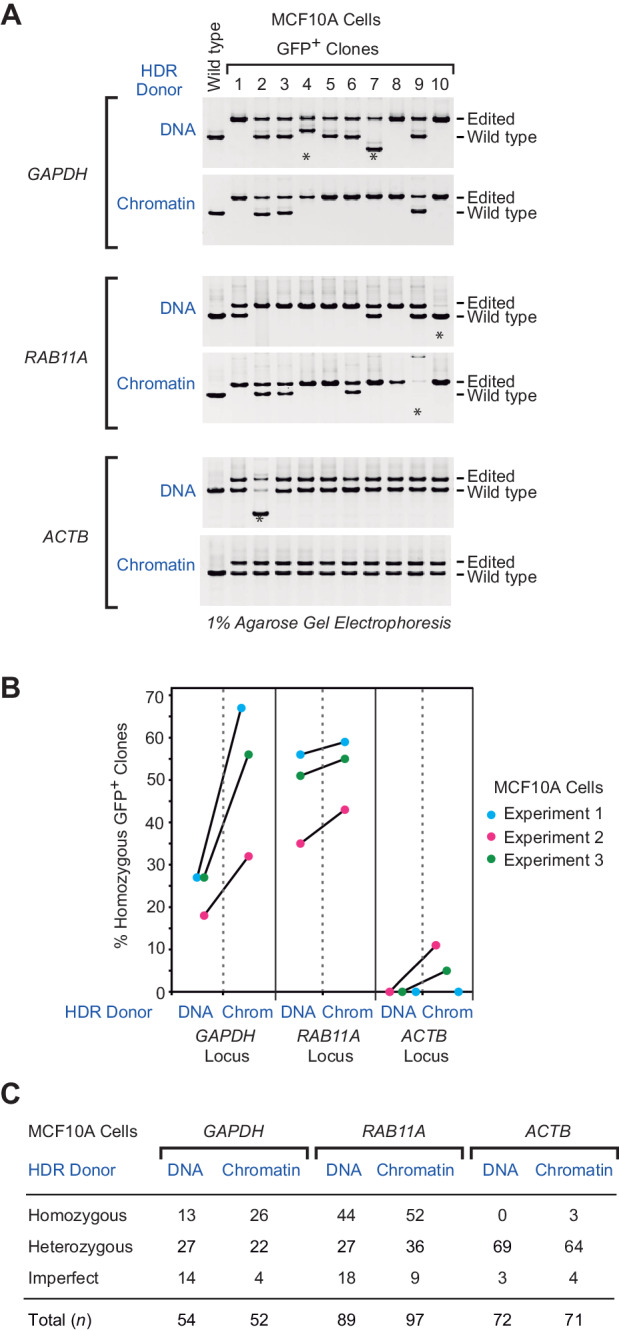
The use of chromatin donor templates increases the efficiency of HDR-mediated homozygous gene editing relative to that seen with DNA donor templates. (**A**) PCR analysis of gDNA from MCF10A GFP-positive clones. Three independent HDR experiments were performed as shown in Figure 1A, and the gDNA from individual GFP-positive clones was analyzed by PCR. The positions of the PCR amplification products from edited and wild-type alleles are indicated. The PCR products derived from control wild-type cells are also included (left lane of each panel). The asterisks indicate imperfect clones that appear to contain at least one improperly edited chromosome, as indicated by either the absence of an edited chromosome or the presence of a PCR product whose size is not consistent with that of an edited or wild-type chromosome. The positions of the primer pairs (F1, R1) in the PCR analysis of each locus are shown in Figure 2—figure supplement 1. The results from a representative subset of the GFP-positive clones are shown. The complete set of PCR results are in Figure 2—figure supplements 2, 3 and 5. (**B**) The percentages of GFP-positive homozygous clones in three independent HDR experiments at each of the target loci. The results from each independent experiment (with DNA versus chromatin donor templates) are denoted with a connector line. The *p*-values were determined by using Welch's t-test. The calculated *p*-values are as follows: *p *= 0.062, *p *= 0.56, and *p *= 0.17 for the *GAPDH*, *RAB11A* and *ACTB* data sets, respectively. (**C**) Summary of the PCR analysis. MCF10A cells are diploid, and each clone was classified as homozygous (with two precisely edited chromosomes), heterozygous (with one precisely edited chromosome and one wild-type chromosome), or imperfect, as defined in A.

For many applications of HDR, it is essential to modify all of the copies of the target gene. Therefore, to test the frequency of occurrence of precise homozygous gene editing in the diploid MCF10A cells, we carried out PCR analyses of the individual GFP-positive clones, and we observed a variable but consistently higher frequency of homozygous HDR insertions with chromatin donor templates than with naked DNA donor templates at all three loci (*GAPDH*, *RAB11A*, and *ACTB*) in MCF10A cells (Figure 2 and Figure 2—figure supplements 1–5). At the *GAPDH* locus, the use of chromatin relative to naked DNA donor templates resulted in a 2.1-fold increase in homozygous editing. At the *RAB11A* locus, there was a high frequency of homozygous insertions with the naked DNA donor template, and the use of a chromatin donor template only slightly augments (1.1-fold increase) the percentage of homozygous clones. Strikingly, at the *ACTB* locus, homozygous insertions were observed only with a chromatin donor template. These findings thus show that the use of chromatin relative to naked DNA donor templates can increase the efficiency of homozygous editing.

**Figure 2—figure supplement 1.**
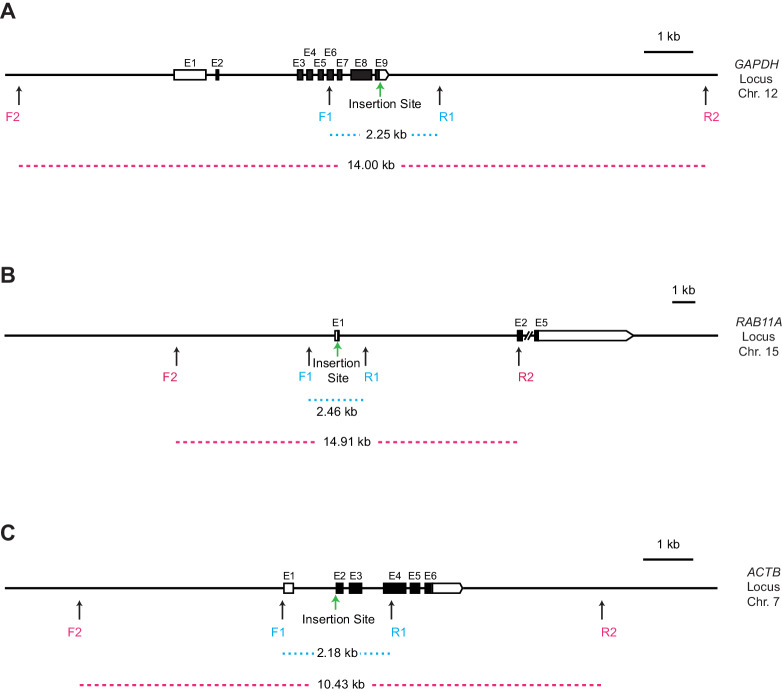
Diagrams of the positions of the primer sets for the PCR analysis of GFP-positive clones at the *GAPDH*, *RAB11A*, and *ACTB* loci. (**A**) *GAPDH* locus. (**B**) *RAB11A* locus. (**C**) *ACTB* locus. The expected PCR product sizes with wild-type gDNA (dashed lines), the positions of the primers (F1, R1, F2, R2; black arrows), and the DNA insertion sites (green arrows) at each locus are indicated. Two primer pairs are shown for each locus: F1, forward primer 1; R1, reverse primer 1; F2, forward primer 2; R2, reverse primer 2. E, Exon. The HDR-mediated insertions increase the lengths of the PCR products by 771 bp, 732 bp, and 730 bp at the *GAPDH*, *RAB11A*, and *ACTB* loci, respectively.

**Figure 2—figure supplement 2.**
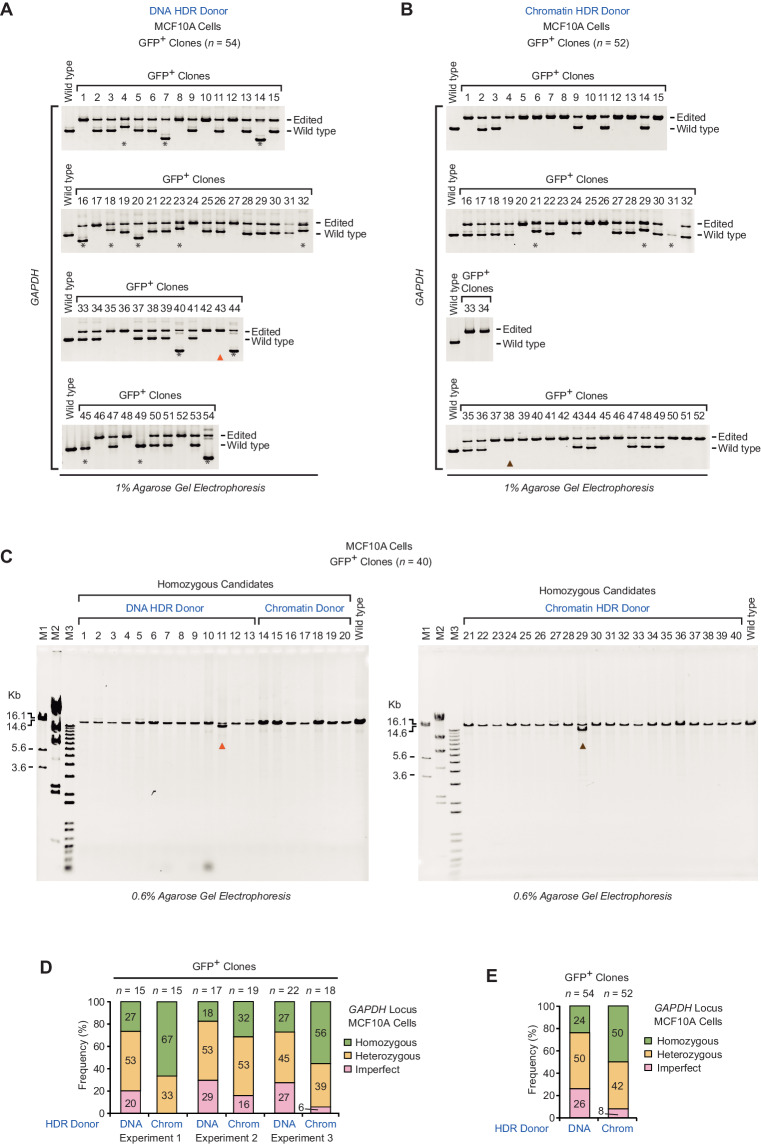
PCR analysis of gDNA from GFP-positive clones at the *GAPDH* locus in MCF10A cells. (**A**) Clones (*n* = 54) collected from three independent HDR experiments with a DNA donor template. Lanes 1 to 15, 16 to 32, and 33 to 54 correspond to experiment 1, experiment 2, and experiment 3, respectively. (**B**) Clones (*n* = 52) collected from three independent HDR experiments with a chromatin donor template. Lanes 1 to 15, 16 to 34, and 35 to 52 correspond to experiment 1, experiment 2, and experiment 3, respectively. In panels A and B, the positions of the PCR amplification products from edited and wild-type alleles are indicated. Asterisks denote imperfect clones. Clones were classified as defined in the figure legend of Figure 2 of the main text. The triangles indicate imperfect clones (as assessed with long-range PCR analysis; see panel C, below) with an apparently homozygous genotype in the standard PCR analysis, as in panels A and B. (**C**) Long-range PCR analysis of homozygous candidate clones (*n* = 40). Clones collected from three independent HDR experiments with either a DNA donor template (lanes 1 to 13) or a chromatin donor template (lanes 14 to 40) were analyzed. These clones were preliminarily classified as homozygous based on the PCR analysis shown in A and B. Clones that have a deletion within a 14.0 kb region surrounding the target insertion site, as indicated by the presence of an additional PCR product that is smaller than that of the properly edited allele, are denoted with triangles. The PCR product (14.0 kb) from gDNA of wild-type cells is also shown. The positions of the primer pairs (F2, R2) for the PCR analyses (panels A–C) are depicted in Figure 2—figure supplement 1A. DNA size markers: M1 (1 kb Plus DNA Ladder, Invitrogen); M2 (λ DNA-HindIII Digest, NEB); M3 (bacteriophage T7 DNA digested with HindIII). (**D**) Frequency of occurrence of homozygous, heterozygous, and imperfect clones in three independent HDR experiments. *n*, number of clones analyzed. (**E**) Summary of the combined results at the *GAPDH* locus in MCF10A cells. The percentages were calculated based on the data for the *GAPDH* locus in Figure 2C.

**Figure 2—figure supplement 3.**
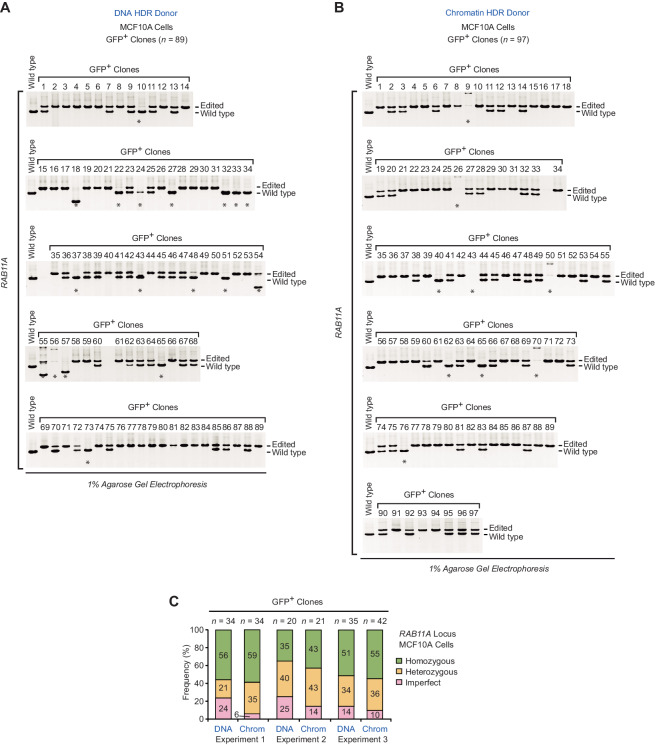
PCR analysis of gDNA from GFP-positive clones at the *RAB11A* locus in MCF10A cells. (**A**) Clones (*n* = 89) collected from three independent HDR experiments with a DNA donor template. Lanes 1 to 34, 35 to 54, and 55 to 89 correspond to experiment 1, experiment 2, and experiment 3, respectively. (**B**) Clones (*n* = 97) collected from three independent HDR experiments with a chromatin donor template. Lanes 1 to 34, 35 to 55, and 56 to 97 correspond to experiment 1, experiment 2, and experiment 3, respectively. In A and B, the positions of the PCR amplification products from edited and wild-type alleles are indicated. Asterisks indicate imperfect clones, as defined in the figure legend of Figure 2. (**C**) Frequency of occurrence of homozygous, heterozygous, and imperfect clones in each of three independent HDR experiments. *n*, number of clones analyzed.

**Figure 2—figure supplement 4.**
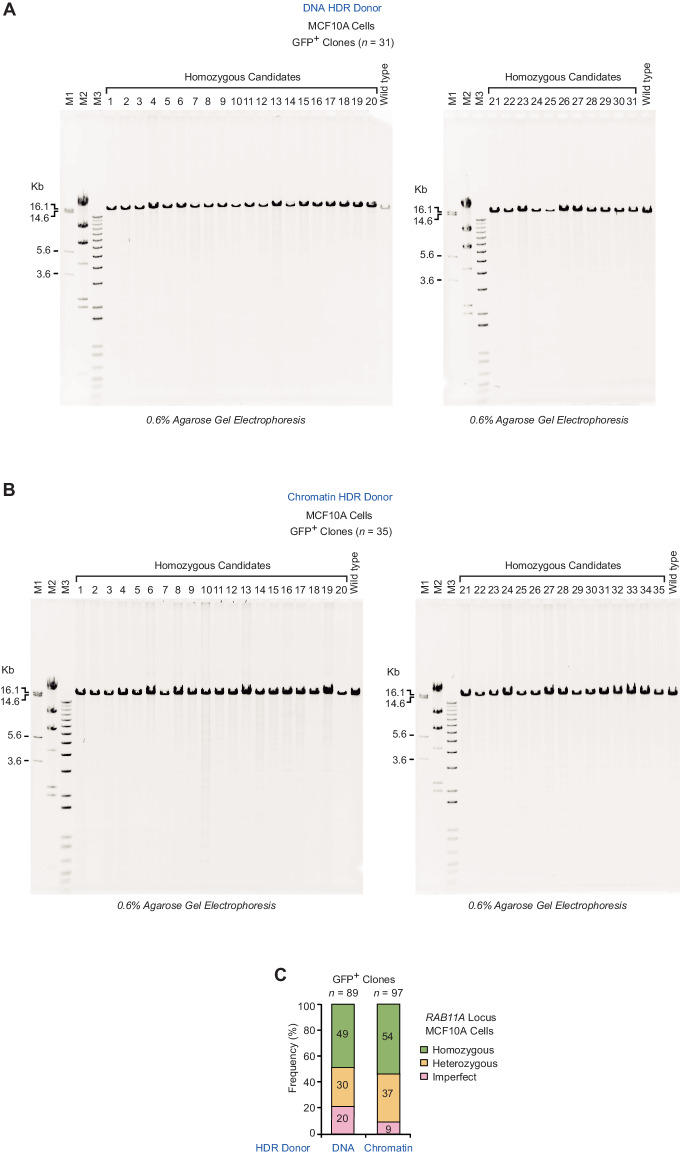
Long-range PCR analysis of gDNA from GFP-positive clones at the *RAB11A* locus in MCF10A cells. (**A**) Analysis of homozygous candidates (*n* = 31) collected from three independent HDR experiments with a DNA donor template. (**B**) Analysis of homozygous candidates (*n* = 35) collected from three independent HDR experiments with a chromatin donor template. In panels A and B, the PCR product (14.91 kb) from gDNA of wild-type cells is also shown. The positions of the primers (F2, R2) in the PCR analysis are depicted in Figure 2—figure supplement 1B. DNA size markers: M1 (1 kb Plus DNA Ladder, Invitrogen); M2 (λ DNA-HindIII Digest, NEB); M3 (bacteriophage T7 DNA digested with HindIII). (**C**) Summary of the combined results at the *RAB11A* locus in MCF10A cells. The percentages were calculated based on the data for the *RAB11A* locus in Figure 2C.

**Figure 2—figure supplement 5.**
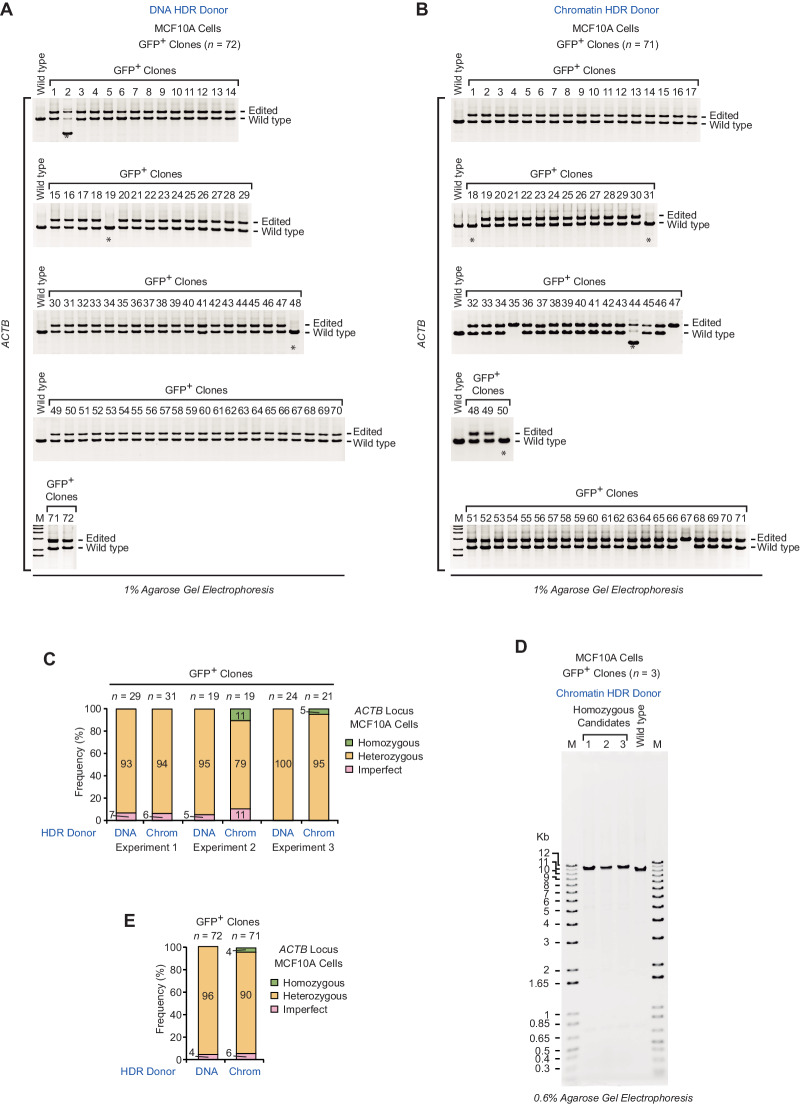
PCR analysis of gDNA from GFP-positive clones at the *ACTB* locus in MCF10A cells. (**A**) Clones (*n* = 72) collected from three independent HDR experiments with a DNA donor template. Lanes 1 to 29, 30 to 48, and 49 to 72 correspond to experiment 1, experiment 2, and experiment 3, respectively. (**B**) Clones (*n* = 71) collected from three independent HDR experiments with a chromatin donor template. Lanes 1 to 31, 32 to 50, and 51 to 71 correspond to experiment 1, experiment 2, and experiment 3, respectively. In A and B, the positions of the PCR amplification products from edited and wild-type alleles are indicated. M, DNA size markers (1.65, 2, 3, 4, 5, 6 kb; 1 kb Plus DNA Ladder, Invitrogen). Asterisks denote imperfect clones as defined in Figure 2. (**C**) Frequency of occurrence of homozygous, heterozygous, and imperfect clones in three independent HDR experiments. *n*, number of clones analyzed. (**D**) Long-range PCR analysis of homozygous candidates collected from HDR experiments with a chromatin donor template. The PCR product (10.43 kb) from gDNA of wild-type cells is also shown. The positions of the primers (F2, R2) in the PCR analysis are depicted in Figure 2—figure supplement 1C. (**E**) Summary of the combined results at the *ACTB* locus in MCF10A cells. The percentages were calculated based on the data for the *ACTB* locus in Figure 2C.

We also observed imperfect editing, in which there was at least one improperly edited chromosome, as indicated by either the absence of an edited chromosome or the presence of a PCR product whose size is not consistent with that of an edited or wild-type chromosome. In addition, by performing long-range PCR as in Kosicki et al., 2018, we identified two apparently homozygous clones that contained one chromosome with a precisely edited allele and one chromosome with a large deletion at the other allele (Figure 2—figure supplement 2). Hence, in the generation of homozygous clones, it is important to carry out both standard and long-range PCR analyses.

The overall efficiency of achieving homozygous editing in diploid MCF10A cells was 15-fold (7.4 × 2.1) at the *GAPDH* locus, 3.2-fold (2.9 × 1.1) at the *RAB11A* locus, and large but not quantifiable at the *ACTB* locus, at which we saw homozygous editing only with a chromatin donor template. The *ACTB* locus serves as an example in which the use of a chromatin template relative to a naked DNA template was the difference between a successful and an unsuccessful HDR experiment.

**Figure 3.**
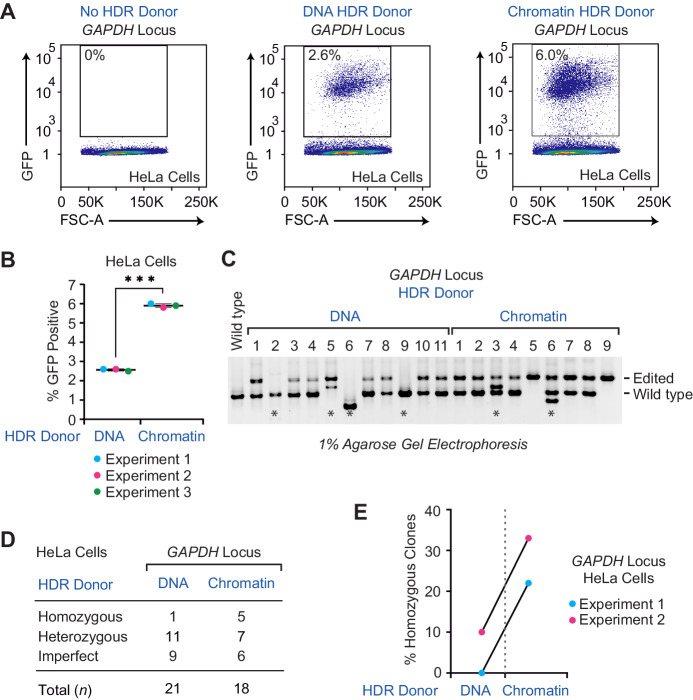
The efficiency of HDR-mediated gene editing with CRISPR-Cas9 is higher with a chromatin donor template than with a DNA donor template in HeLa cells. (**A**) The use of a chromatin donor template relative to a naked DNA donor template results in an increase of GFP-positive cells. HDR experiments were performed as depicted in Figure 1A with HeLa cells and the *GAPDH* locus donor template. The population of GFP-positive cells was gated based on control cells that show no GFP expression (no HDR donor; left panel). Representative data from one out of three independent experiments are shown. The results of the other two biological replicates are in Figure 3—figure supplement 1. The percentage of GFP-positive cells is indicated in each plot. FSC-A: forward scatter area. (**B**) Individual results of flow cytometry analysis from three independent experiments with the *GAPDH* locus and HeLa cells. The data points from each independent experiment are designated with the same colored dots. The *p*-value was determined by using Welch's t-test. ***, *p *<0.0001. The mean and standard deviation are indicated. (**C**) The use of a chromatin HDR donor template results in an increase in the efficiency of homozygous edited clones relative to that seen with a DNA donor template. PCR analysis of edited genomic DNA was carried out as in Figure 2A. The positions of the PCR amplification products from edited and wild-type chromosomes are shown. The PCR products from control wild-type cells are also included (left lane). The results from a representative subset of the GFP-positive clones are shown. The results from the other GFP-positive clones that were analyzed are in Figure 3—figure supplement 2. (**D**) Summary of the PCR analysis of clones obtained in the HDR-mediated insertion of GFP sequences at the *GAPDH* locus in HeLa cells. The homozygous clones have four copies of the integrated GFP sequence, the heterozygous clones have one to three copies of the integrated GFP sequence, and the imperfect clones appear to contain improperly edited chromosomes, as indicated by either the absence of an edited chromosome or the presence of a PCR product whose size is not consistent with that of an edited or wild-type chromosome. (**E**) The percentages of GFP-positive homozygous clones in two independent HDR experiments. The results from each independent experiment (with DNA versus chromatin donor templates) are denoted with a connector line.

To determine whether a chromatin donor template affects the efficiency of HDR in a different cell line, we examined the insertion of GFP sequences at the *GAPDH* locus in HeLa cells, which are human cervical adenocarcinoma cells that are widely used in biomedical research. HeLa cells are aneuploid and contain four copies of the *GAPDH* gene, which is located on chromosome 12. In these experiments, we observed that the use of a chromatin donor template results in a 2.3-fold increase (average of three biological replicates) in the efficiency of insertion of the GFP sequence in at least one *GAPDH* locus in HeLa cells (Figure 3A, B and Figure 3—figure supplement 1). We then examined the formation of homozygous edited clones that are generated upon targeted insertion of the GFP sequence at all four copies of the *GAPDH* locus in HeLa cells. In this analysis, we found a substantial increase (5/18 clones versus 1/21 clones) in the efficiency of formation of homozygous clones with the use of a chromatin donor template instead of a naked DNA donor template (Figure 3C,D,E and Figure 3—figure supplement 2). Hence, these results show a strong enhancement of HDR by using a chromatin relative to a naked DNA donor template in HeLa cells.

**Figure 3—figure supplement 1.**
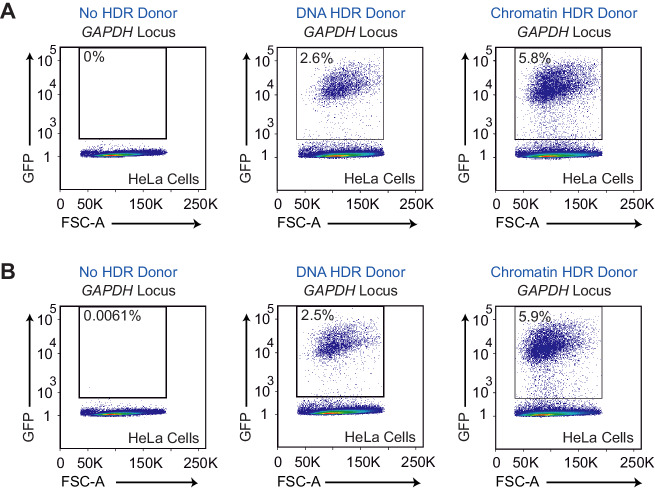
Flow cytometry analyses of biological replicates of HDR-mediated gene integration experiments in HeLa cells. (**A**) Data from HDR experiment 2. (**B**) Data from HDR experiment 3. HDR experiments were performed as outlined in Figure 1A. GFP-positive cells was gated based on cells that show no GFP expression (no HDR donor; left panels).

**Figure 3—figure supplement 2.**
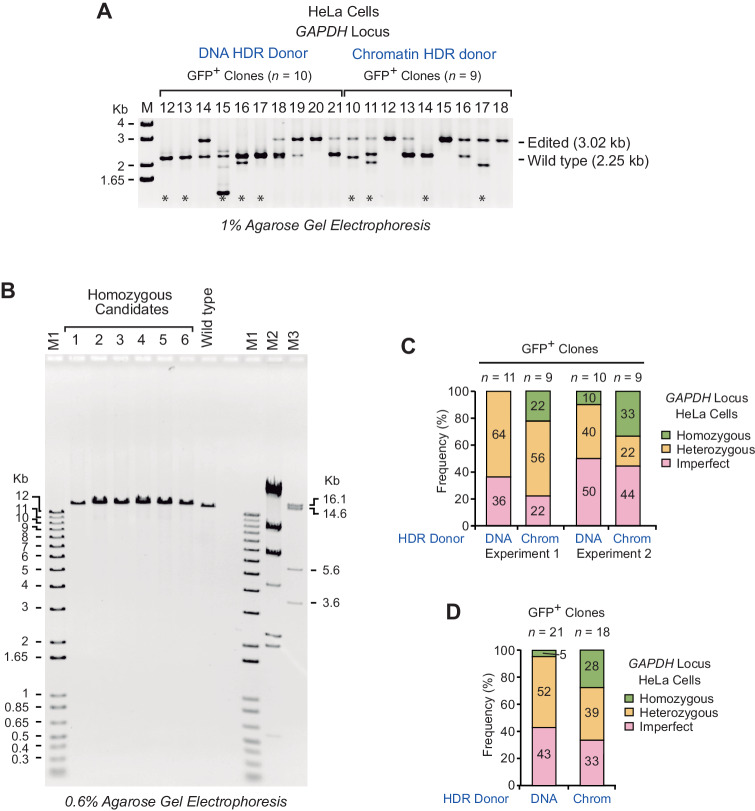
PCR analysis of gDNA from GFP-positive clones in HeLa cells. (**A**) Clones collected from HDR experiments with a DNA donor template (clones 12 to 21) or a chromatin donor template (clones 10 to 18). The positions of the PCR products of the wild-type and HDR-edited alleles are indicated. The positions of the primer pairs (F1, R1) are depicted in Figure 2—figure supplement 1A. The asterisks denote imperfect clones, as specified in the figure legend of Figure 2. M, DNA size marker (1 kb DNA ladder, Invitrogen). (**B**) Long-range PCR analysis of six homozygous clones collected from two independent HDR experiments. The PCR product (14.0 kb) from gDNA of wild-type cells is also shown. The positions of the primer pairs (F2, R2) are depicted in Figure 2—figure supplement 1A. DNA size markers: M1 (1 kb Plus DNA Ladder, Invitrogen); M2 (λ DNA-HindIII Digest, NEB); M3 (bacteriophage T7 DNA digested with HindIII). (**C**) Frequency of occurrence of homozygous, heterozygous, and imperfect clones in two independent HDR experiments. *n*, number of clones analyzed. (**D**) Summary of the combined results at the *GAPDH* locus in HeLa cells. The percentages were calculated based on the data in Figure 3D. *n*, number of clones analyzed.

**Figure 3—figure supplement 3.**
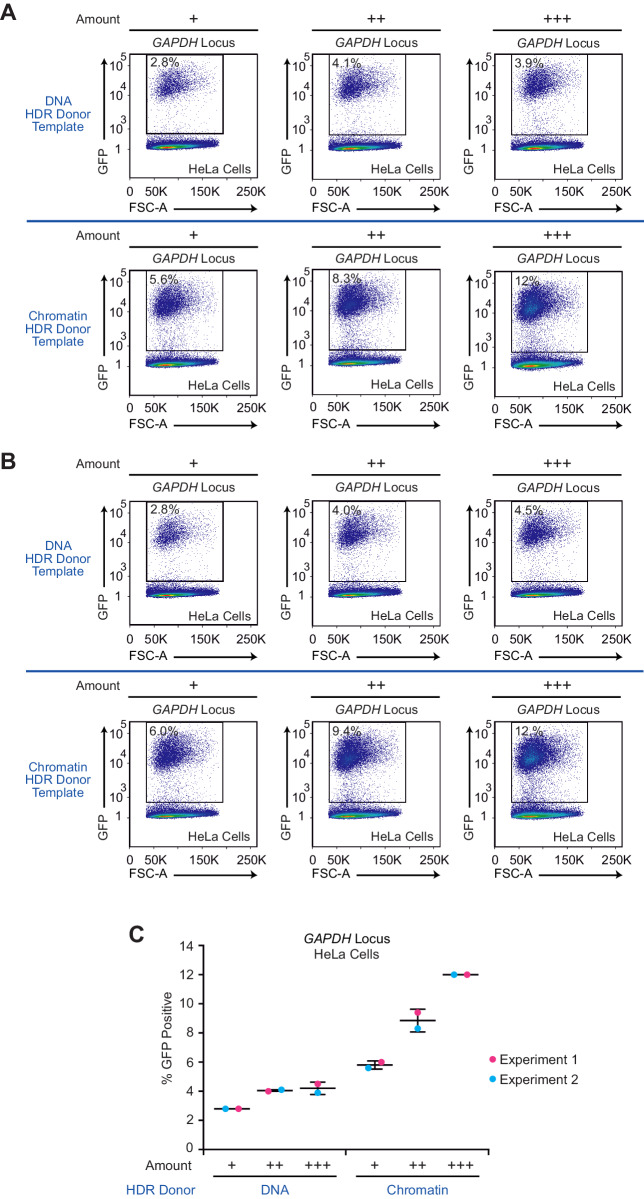
The efficiency of GFP insertion with different amounts of donor template in HeLa cells is higher with chromatin than with DNA. (**A**) The results from HDR experiment 1. (**B**) The results from HDR experiment 2. In A and B, the experiments were performed as depicted in Figure 1A. HeLa cells were co-transfected with the Cas9-T2A-mCherry plasmid containing the sgRNA sequence targeting the *GAPDH* locus and 0.625 µg (+), 1.25 µg (++), or 1.88 µg (+++) of the corresponding HDR donor template as either DNA or chromatin. As a reference, we used 1.25 µg (++) of donor template as DNA or chromatin in our standard experiments, such as those shown in the main figures. At 24 hr post-transfection, mCherry-positive cells were enriched by FACS and cultured for an additional 10 days. The expression of GFP was then analyzed by flow cytometry. (**C**) Summary of the results from HDR experiments 1 and 2. The percentages of GFP-positive cells in each experiment are shown. The mean and standard deviation (horizontal bars) are depicted for each experimental condition (*n* = 2).

**Figure 3—figure supplement 4.**
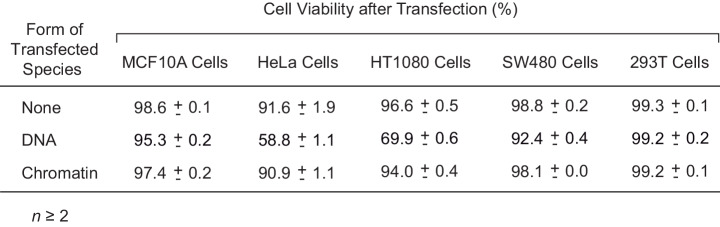
Chromatin templates are of comparable or lower toxicity to cells relative to naked DNA templates. Cell viability after transfection with a 3 kb plasmid as either naked DNA or chromatin was determined along with the viability of mock-transfected (no DNA or chromatin) cells. The cell viability was assessed by flow cytometry in the presence of DAPI (4',6-diamidino-2-phenylindole). The analysis was performed 48 hr after transfection. The mean and standard deviation from at least two independent experiments with each cell line are shown.

We additionally tested the effect of varying the amount of donor template DNA (as chromatin or naked DNA) upon the efficiency of HDR (Figure 3—figure supplement 3). To this end, we used 0.5, 1.0, and 1.5 times the mass of DNA as in a standard experiment with the *GAPDH* donor template in HeLa cells. At each of the three amounts of donor template, we consistently saw a higher efficiency of generation of GFP-positive cells with chromatin relative to naked DNA. Moreover, there was an increase in the fold-enhancement by chromatin as the amount of donor template was increased. We thus observed that a chromatin donor template functions better than a naked DNA donor template for HDR at different concentrations.

Because chromatin has rarely been used in cell transfection experiments, we also investigated the toxicity of chromatin relative to naked DNA in five different human cell lines (Figure 3—figure supplement 4). These experiments revealed that chromatin is of comparable or lower toxicity to cells relative to naked DNA in transfection experiments. This low toxicity of chromatin to cells could be useful for HDR applications in which there is low cell viability after transfection.

## Discussion

Here we show that the efficiency of HDR-mediated gene editing can be increased by using a chromatin donor template instead of a naked DNA donor template. Why is chromatin more effective as an HDR donor template than naked DNA? We suggest that chromatin, as the natural form of DNA in the eukaryotic nucleus, is the preferred substrate (relative to naked DNA) for the factors that mediate homologous recombination in cells. In previous biochemical studies, we and others found that eukaryotic Rad51 and Rad54, but not bacterial RecA, can mediate homologous strand pairing, an early step in HDR, with a chromatin donor template (Alexiadis and Kadonaga, 2002; Jaskelioff et al., 2003). Moreover, we observed that homologous strand pairing occurs more efficiently with a chromatin donor template than with a naked DNA donor template (Alexiadis and Kadonaga, 2002). Hence, the new findings on HDR with chromatin donor templates in cells are consistent with the results of the earlier biochemical studies on homologous strand exchange.

In general, a wide range of efficiencies of HDR has been observed in different cell types and with different methodologies. A common factor in these HDR experiments has been, however, the use of a non-chromatin donor template. In this work, we sought to focus specifically on directly comparing the relative efficiencies of HDR with chromatin versus naked DNA donor templates. In these experiments, we consistently observed a higher efficiency of HDR with chromatin relative to naked DNA. These effects include the increased efficiency of targeted insertion of GFP sequences in both loci of a diploid chromosome and in all loci of a tetraploid chromosome. These findings therefore suggest that the use of a chromatin donor template instead of a naked DNA donor template would be a broadly useful strategy for the precise insertion or replacement of DNA sequences via HDR with different methods. Moreover, transfection of chromatin donor templates, which can be simply prepared by salt dialysis methodology with purified DNA and core histones, does not affect cell viability. Thus, current methods for HDR can be easily adapted to include chromatin donor templates in place of their naked DNA counterparts.

In this regard, it is notable that we reconstituted chromatin by using native core histones from *Drosophila* embryos. These histones contain an undefined broad mixture of covalent histone modifications (Levenstein and Kadonaga, 2002). Because the core histones and their modifications are highly conserved throughout eukaryotes, it seems likely that similar results would be obtained with core histones from other sources. It is possible, however, that the magnitude of enhancement of HDR by chromatin could be further increased by variation of the core histone sequences and modifications.

In conclusion, although there are excellent techniques for the alteration of short (<50 bp) stretches of DNA (Rees and Liu, 2018; Molla and Yang, 2019; Anzalone et al., 2019), there remains a need for increasing the efficiency of the specific insertion or replacement of longer DNA segments that may contain sequences such as the coding regions of genes. We anticipate that chromatin donor templates might be particularly useful for such applications. In addition, we expect that many new gene editing techniques will be developed in the future, and that some of these methods will benefit from the use of chromatin donor templates. Furthermore, the low toxicity of chromatin to cells may be useful for many current and future methods. There is considerable potential to the use of the natural form of the donor template in gene editing experiments. It is our hope that these findings will advance the utility of precise genome editing in basic, translational, and clinical research.

## Materials and methods

To ensure the reproducibility of the results, at least two biological replicates were performed for each experimental condition. The exact number of replicates of each experiment is indicated in its associated figure legend.

### DNA constructs

CRISPR RNA (crRNA) sequences targeting the *GAPDH*, *RAB11A*, or *ACTB* loci were each inserted into the pU6-(BbsI)CBh-Cas9-T2A-mCherry vector (Addgene plasmid # 64324) as described (Ran et al., 2013). The crRNA sequences that were used are as follows: *GAPDH*, GAGAGAGACCCTCACTGCTG; *RAB11A*, GGTAGTCGTACTCGTCGTCG; *ACTB*, GGTGAGCTGCGAGAATAGCC. The donor template plasmid for the modification of the *GAPDH* locus was generated as follows. Two homology arm (HA) sequences (~1 kb each) were PCR-amplified with Phusion polymerase (NEB) and genomic DNA (gDNA) from HeLa cells. The oligonucleotides that were used are as follows (the upper case letters are complementary to *GAPDH* or T2A-EGFP sequences): 5' HA, agagataagcttGGACACGCTCCCCTGACTT, agagatggatccCTCCTTGGAGGCCATGTGGG; 3' HA, tgatagggtaccCCTGCCACACTCAGTCCC, tgataggaattcGCTGGGGTTACAGGCGTGCG. The T2A-EGFP sequence was PCR-amplified from the PX461 plasmid (Addgene plasmid # 48140) with the following oligonucleotides: agagatggatccGAGGGCAGAGGAAGTCTGCT and agagatggtaccTTACTTGTACAGCTCGTCCA. Then, the three DNA fragments were sequentially subcloned into the pBluescript KS vector (Stratagene). The 3' HA sequence was inserted between the KpnI and EcoRI sites; the T2A-EGFP sequence was inserted between the BamHI and the KpnI sites; and the 5' HA sequence was inserted between the HindIII and the BamHI sites. All restriction enzymes were from NEB. The donor template plasmid for the modification of the *RAB11A* locus was Addgene plasmid # 112012, and the donor template plasmid for the modification of the *ACTB* locus was Addgene plasmid # 87425.

### Chromatin reconstitution

Native *Drosophila* core histones from embryos collected from 0 to 12 hr after egg deposition were purified as described (Fyodorov and Levenstein, 2002; Khuong et al., 2017). The donor repair template plasmids were purified with the HiSpeed plasmid kit (Qiagen). The optimal histone:DNA ratio for each donor repair template was determined by carrying out a series of reactions with different histone:DNA ratios and then assessing the quality of chromatin by the micrococcal nuclease digestion assay, as described (Fyodorov and Levenstein, 2002; Khuong et al., 2017). Chromatin was reconstituted with purified core histones by using the salt dialysis method (Stein, 1989; Fei et al., 2015). In a typical chromatin reconstitution reaction, 50 µg plasmid DNA and 50 µg core histones were combined in TE buffer (10 mM Tris-HCl, pH 8, containing 1 mM EDTA) containing 1 M NaCl in a total volume of 150 µL. The mixture was dialyzed at room temperature against the following buffers in the indicated order: 2 hr in TE containing 0.8 M NaCl; 3 hr in TE containing 0.6 M NaCl; 2.5 hr in TE containing 50 mM NaCl. The quality of the resulting chromatin was assessed by using the micrococcal nuclease digestion assay, and the chromatin was stored at 4°C until use.

### Cell lines

HeLa cells were a gift from Dr. Anjana Rao (La Jolla Institute for Immunology). MCF10A cells were a gift from Dr. Jichao Chen (The University of Texas MD Anderson Cancer Center). The MCF10A and HeLa cells were not authenticated. The MCF10A cells and HeLa cells were tested for mycoplasma and found to be negative for mycoplasma contamination.

### Cell culture

MCF10A cells (non-tumorigenic mammary epithelial cells) were maintained in DMEM/F-12 medium (Gibco) supplemented with 20 ng/mL EGF, 500 ng/mL hydrocortisone (Sigma), 10 μg/mL insulin (Sigma), 100 ng/mL cholera toxin (Sigma), 100 U/mL penicillin and 100 µ/mL streptomycin (Gibco), and 5% horse serum (Gibco) at 37°C and 5% CO_2_. HeLa cells (human cervical carcinoma cells), HT1080 cells (human fibrosarcoma cells), SW480 cells (human colorectal adenocarcinoma cells), and 293 T cells (derived from primary human embryonic kidney cells) were maintained in DMEM, high glucose medium (Corning) supplemented with 10% fetal bovine serum (Gibco) and 100 U/mL penicillin and 100 µ/mL streptomycin (Gibco) at 37°C and 5% CO_2_.

### Cell transfection

In each series of experiments, cell transfections with chromatin or DNA donor templates were performed by following standard protocols under exactly the same conditions. Transfection of HeLa cells was performed with Lipofectamine 3000 (Invitrogen) according to the manufacturer's recommendations. Linear polyethylenimine (PEI 25K; 25,000 MW; Polysciences, Inc) was used for transfection of MCF10A cells at a PEI:DNA mass ratio of 3:1. The transfections were performed as follows. 5 × 10^5^ cells/well were plated in six well plates the day before transfection. For each CRISPR-Cas9 target locus, cells were co-transfected with equal amounts of the target-specific donor repair template (as free plasmid DNA or chromatin) and the Cas9 coding plasmid containing the target-specific single guide RNA sequence. For HeLa cells, DNA (1.25 µg) or chromatin (containing 1.25 µg of DNA) was used in each transfection (except for the experiment in Figure 3—figure supplement 3, in which 1.25 µg of the Cas9 coding plasmid containing the single guide targeting the *GAPDH* locus was co-transfected with 0.625 µg, 1.25 µg, or 1.875 µg of donor template DNA as naked DNA or chromatin); for MCF10A cells, DNA (1.5 µg) or chromatin (containing 1.5 µg of DNA) was used in each transfection.

### FACS and flow cytometry analysis

At 24 hr post-transfection, cells were detached with 0.25% trypsin (Corning). After centrifugation, the cell pellets were resuspended in culture media containing 250 ng/mL DAPI (Sigma). mCherry-positive, DAPI-negative cells were sorted by FACS and collected in six well plates (HeLa cells; 100,000 cells/well) or 24 well plates (MCF10A cells; 30,000 cells/well). Then, the cells were passaged twice before the analysis of the expression of GFP by flow cytometry. GFP-positive single-cells were sorted by FACS into 96 well plates. To determine the percentage of GFP-positive cells, at least 100,000 cells of each condition were analyzed by flow cytometry with a BD FACSAria Fusion or a BD FACSAria2 instrument at the Human Embryonic Stem Cell Core Facility (UCSD). The BD FACSDiva Software was used for data acquisition, and data analysis was performed with FlowJo version 10.6.1 (BD).

### Molecular analysis of the targeted loci

Genomic DNA samples from wild-type cells as well as from independent GFP-positive clones were isolated with the Quick Extract DNA extraction solution (Lucigen) by following the manufacturer's recommendations, and were then subjected to PCR analysis. First, the occurrence of edited alleles was analyzed with primers that flank the 5' and 3' homology arm sequences (and thus do not contain sequences in the donor template) at the location in which the GFP DNA was inserted. The specific primers that were used are as follows: *GAPDH*, F1: TGACAACAGCCTCAAGATCATCAGG, R1: GATGGAGTCTCATACTCTGTTGCCT; *RAB11A*, F1: TGGGAAGTGGACATCATTGG, R1: GACCCTCCAATATGTTCTGT; *ACTB*, F1: AATGCTGCACTGTGCGGCGA, R1: ATGGCATGGGGGAGGGCATA. Then, genomic DNA from potentially homozygous GFP-positive clones was analyzed by long-range PCR analysis with LongAmp Hot Start *Taq* DNA Polymerase (NEB), as described by Kosicki et al., 2018. The primers that were used are as follows. *GAPDH*, F2: CTCCTGCAGTGATTTGTTTCTTCTT, R2: ACTCATTCTCCCAACACACATCAAA; *RAB11A*, F2: GCTTTATCTTCTTTTTGCTCACCTG, R2: GTGTCCCATATCTGTGCCTTTATTG; *ACTB*, F2: ATGAATAAAAGCTGGAGCACCCAA, R2: TTGTGCAGCTATACGCAAGATTAAG. The locations of the PCR primers at the *GAPDH*, *RAB11A*, and *ACTB* loci are depicted in Figure 2—figure supplement 1. To confirm the integrity of the homozygous clones obtained with chromatin donor templates, we determined the DNA sequences of three *GAPDH* clones and three *ACTB* clones across the insertion junctions and found that the GFP sequences were precisely inserted into the target sites in all six clones.

### Statistical analysis

The two-tailed Welch t-test with alpha = 0.05 was performed by using GraphPad Prism version 8.4.1 (GraphPad Software).

## Data Availability

All data generated or analysed during this study are included in the manuscript and supporting files.

## Additional files

Transparent reporting form
