# Supplementary material for: Enhancement of homology-directed repair with chromatin donor templates in cells
Source: eLife. 2020 Apr 28;9:e55780. doi: 10.7554/eLife.55780 (PMC7244325; doi:10.7554/eLife.55780)
Supplement: Transparent reporting form [file elife-55780-transrepform.doc]

***eLife’s* transparent reporting form**

We encourage authors to provide detailed information *within their submission* to facilitate the interpretation and replication of experiments. Authors can upload supporting documentation to indicate the use of appropriate reporting guidelines for health-related research (see [EQUATOR Network](http://www.equator-network.org/ )), life science research (see the [BioSharing Information Resource](https://biosharing.org/)), or the [ARRIVE guidelines](http://www.plosbiology.org/article/info:doi/10.1371/journal.pbio.1000412) for reporting work involving animal research. Where applicable, authors should refer to any relevant reporting standards documents in this form.

If you have any questions, please consult our Journal Policies and/or contact us: [editorial@elifesciences.org](mailto:editorial@elifesciences.org).

**Sample-size estimation**

- You should state whether an appropriate sample size was computed when the study was being designed
- You should state the statistical method of sample size computation and any required assumptions
- If no explicit power analysis was used, you should describe how you decided what sample (replicate) size (number) to use

Please outline where this information can be found within the submission (e.g., sections or figure legends), or explain why this information doesn’t apply to your submission:

The number of replicates is similar to that of published work that is related to our study. The number of biological replicates of each experiment is given in the figure legends.

**Replicates**

- You should report how often each experiment was performed
- You should include a definition of biological versus technical replication
- The data obtained should be provided and sufficient information should be provided to indicate the number of independent biological and/or technical replicates
- If you encountered any outliers, you should describe how these were handled
- Criteria for exclusion/inclusion of data should be clearly stated
- High-throughput sequence data should be uploaded before submission, with a private link for reviewers provided (these are available from both GEO and ArrayExpress)

Please outline where this information can be found within the submission (e.g., sections or figure legends), or explain why this information doesn’t apply to your submission:

Definitions of biological and technical replicates:

(taken from the NIH)

https://www.nigms.nih.gov/training/documents/module3-biological-and-technical-replicates.pdf

-Biological replicates are parallel measurements of biologically distinct samples that capture random biological variation, which may itself be a subject of study or a source of noise.

-Technical replicates are repeated measurements of the same sample that represent independent measures of the random noise associated with protocols or equipment.

In this study, each experiment was performed a minimum of two independent times with different preparations of donor template in the form of DNA or chromatin. Because these experiments were performed with different preparations of donor template (i.e., "biologically distinct samples"), we consider these experiments to be biological replicates.

In the paper, we have included this information at the beginning of the Materials and methods section. It is stated “To ensure the reproducibility of the results, at least two biological replicates were performed for each experimental condition. The exact number of replicates of each experiment is indicated in its associated figure legend."

**Statistical reporting**

- Statistical analysis methods should be described and justified
- Raw data should be presented in figures whenever informative to do so (typically when N per group is less than 10)
- For each experiment, you should identify the statistical tests used, exact values of N, definitions of center, methods of multiple test correction, and dispersion and precision measures (e.g., mean, median, SD, SEM, confidence intervals; and, for the major substantive results, a measure of effect size (e.g., Pearson's r, Cohen's d)
- Report exact p-values wherever possible alongside the summary statistics and 95% confidence intervals. These should be reported for all key questions and not only when the p-value is less than 0.05.

Please outline where this information can be found within the submission (e.g., sections or figure legends), or explain why this information doesn’t apply to your submission:

The statistical analysis is described in the Materials and methods section. All raw data are shown. The number of independent experiments, statistical test method, and *p*-values are indicated in the figure legends as follows.

Figure 1: “(**C**) Individual results from three independent experiments with each of the target loci. The data points from each independent experiment are designated with the same colored dots. The mean and standard deviation are indicated for each set of experiments. The *p*-values were determined by using Welch's t test. **, *p* < 0.01; *, *p* < 0.05. The calculated *p*-values are as follows: *p* = 0.0062 for the *GAPDH* data set; *p* = 0.017 for the *RAB11A* data set; *p* = 0.048 for the *ACTB* data set. (**D**) The use of chromatin relative to naked DNA donor templates results in a 2.3- to 7.4-fold enhancement of GFP-positive cells. The data for each of three independent HDR experiments with each locus are shown. The bars represent mean and standard deviation for each locus.”

Figure 2: “(**B**) The percentages of GFP-positive homozygous clones in three independent HDR experiments at each of the target loci. The results from each independent experiment (with DNA versus chromatin donor templates) are denoted with a connector line. The p-values were determined by using Welch's t-test. The calculated *p*-values are as follows: *p* = 0.062, *p* = 0.56, and *p* = 0.17 for the GAPDH, RAB11A and ACTB data sets, respectively.”

Figure 3: “(**B**) Individual results of flow cytometry analysis from three independent experiments with the *GAPDH* locus and HeLa cells. The data points from each independent experiment are designated with the same colored dots. The *p*-value was determined by using Welch's t-test. ***, *p* < 0.0001. The mean and standard deviation are indicated.”

Figure 3 – figure supplement 3: “(**C**) Summary of the results from HDR experiments 1 and 2. The percentages of GFP-positive cells in each experiment are shown. The mean and standard deviation (horizontal bars) are depicted for each experimental condition (n = 2). The average and standard deviation from two independent experiments are shown.”

Figure 3 – figure supplement 4: “The mean and standard deviation from at least two independent experiments with each cell line are shown.”

(For large datasets, or papers with a very large number of statistical tests, you may upload a single table file with tests, Ns, etc., with reference to sections in the manuscript.)

**Group allocation**

- Indicate how samples were allocated into experimental groups (in the case of clinical studies, please specify allocation to treatment method); if randomization was used, please also state if restricted randomization was applied
- Indicate if masking was used during group allocation, data collection and/or data analysis

Please outline where this information can be found within the submission (e.g., sections or figure legends), or explain why this information doesn’t apply to your submission:

N/A. This study did not involve the allocation of samples into groups.

**Additional data files (“source data”)**

- We encourage you to upload relevant additional data files, such as numerical data that are represented as a graph in a figure, or as a summary table
- Where provided, these should be in the most useful format, and they can be uploaded as “Source data” files linked to a main figure or table
- Include model definition files including the full list of parameters used
- Include code used for data analysis (e.g., R, MatLab)
- Avoid stating that data files are “available upon request”

Please indicate the figures or tables for which source data files have been provided:

N/A. All of the raw data are included in the figures and figure supplements. There are no additional data files.
